# Is combined Total colectomy with posterior Rectopexy effective for internal prolapse and colonic inertia?

**DOI:** 10.1016/j.ijscr.2025.111573

**Published:** 2025-06-26

**Authors:** Mohammadsadra Shamohammadi, Foolad Eghbali

**Affiliations:** a Gastrointestinal and Liver Diseases Research Center, Iran University of Medical Sciences, Tehran, Iran; b Department of Surgery, Rasool-E Akram Hospital, School of Medicine, Iran University of Medical sciences, Tehran, Iran

Dear Editor,

I read with interest the case series by Tabatabaei et al. [1], which evaluates the combined use of total laparoscopic colectomy with posterior suture rectopexy for internal rectal prolapse and colonic inertia. This dual pathology poses a significant therapeutic challenge and is typically managed with separate surgical interventions.

The authors provide valuable preliminary data suggesting symptomatic improvement after the combined procedure, as evidenced by reductions in Wexner constipation scores and improved stool consistency. Their detailed description of the surgical technique and perioperative management adds practical insights. It is commendable that the authors adhered to the updated SCARE guidelines [2], thereby enhancing transparency and reproducibility in surgical case reports.

However, several limitations warrant attention. The small sample size (*n* = 9) and short-term follow-up (6 months) restrict the strength and generalizability of the findings. Moreover, functional outcomes related to defecation, ideally assessed with dynamic imaging such as defecography or MRI, were not reported. Previous studies underscore the importance of these modalities in evaluating surgical success in pelvic floor disorders [3,4].

Additionally, the study lacks comparison with other surgical approaches such as ventral mesh rectopexy combined with colectomy. Current guidelines from the American Society of Colon and Rectal Surgeons (ASCRS) recommend colectomy for refractory colonic inertia and ventral rectopexy for rectal prolapse, though evidence on combining these procedures remains limited [5]. Regarding surgical technique, ventral mesh rectopexy (VMR) has gained popularity due to its low recurrence rates and favorable functional outcomes. However, current meta-analyses do not conclusively demonstrate statistically significant differences in recurrence rates between VMR and posterior sutured rectopexy (PSR) [6]. Furthermore, functional outcomes appear comparable between these approaches at medium-term follow-up [7]. Thus, the choice between VMR and PSR should be individualized, considering surgeon expertise and patient factors.

Despite these limitations, this case series fills an important gap by providing preliminary evidence for a combined surgical strategy addressing both colonic inertia and internal prolapse. Further prospective studies with larger cohorts, longer follow-up, and direct comparisons are essential to validate safety and long-term efficacy.

In conclusion, Tabatabaei et al.'s case series offers meaningful preliminary evidence supporting a combined surgical approach for patients with coexisting internal prolapse and colonic inertia. Future prospective studies with larger populations, longer follow-up, functional outcome assessments, and comparative analyses with alternative techniques such as VMR are necessary to establish the optimal surgical strategy.

## Ethical approval

This letter does not involve original research or patient data, and therefore does not require ethical approval.

## Funding

No funding was received for the preparation of this letter.

## Declaration of Generative Al and Al-assisted technologies in the writing process

These tools were utilized exclusively to refine the manuscript's language and readability.

## Declaration of competing interest

The author declares no competing interests related to this letter.
